# Biochemical parallels between catabolic pathways for lignin-associated aromatic dimers

**DOI:** 10.1128/aem.00276-25

**Published:** 2026-02-18

**Authors:** Joshua K. Michener

**Affiliations:** 1 Biosciences Division, Oak Ridge National Laboratory 6146, Oak Ridge, Tennessee, USA; The Pennsylvania State University, University Park, Pennsylvania, USA

**Keywords:** lignin, *Sphingomonas*

## Abstract

Lignin is one of the most common biopolymers on Earth. In nature, lignin is primarily deconstructed by fungi into mixtures of aromatic compounds that are then assimilated by bacteria and fungi. Industrially, lignin is primarily generated as a byproduct of pulp and paper production and burned for process heat. However, if the appropriate assimilatory pathways were identified, deconstructed lignin could be funneled into value-added products using engineered bacteria. Foundational work has described pathways for assimilation of diverse monomeric aromatic compounds such as protocatechuate, ferulate, and syringate, as well as select dimers including those with β-O-4 and 5-5 interunit linkages. Recent advances have elucidated additional pathways for dimer assimilation, including pathways for new substrates as well as parallel pathways for previously characterized substrates. Comparing these dimer assimilation pathways can illuminate the underlying biochemical logic of assimilation for lignin-associated aromatic dimers and provide opportunities for metabolic engineering to enhance lignin valorization.

## MOTIVATION AND CHALLENGES IN MICROBIAL LIGNIN ASSIMILATION

**Fig 1:**
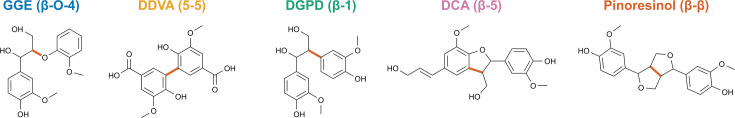
Model lignin-associated aromatic dimers containing representative inter-unit linkages (highlighted in red). DCA, dehydrodiconiferyl alcohol; DDVA, dehydrodivanillic acid; DGPD, diguaiacylpropanediol; GGE, guaiacylglycerol-β-guaiacyl ether.

Lignin is one of the main components of plant biomass, comprising up to 30% of dry biomass by weight (1). It is a complex heterogeneous aromatic polymer formed predominantly from three aromatic monomers polymerized by untemplated radical coupling (2). As a result, lignin contains a variety of interunit linkages in proportions that vary based on complex interactions between genetic and environmental factors (Fig. 1).

Due to this complexity, converting lignin into valuable products is extremely challenging. Select fungi can deconstruct lignin to a mixture of monomers and oligomers, but the process is slow and difficult to scale (3, 4). Thermochemical depolymerization of lignin, such as base-catalyzed depolymerization (5) or reductive catalytic fractionation (6), readily breaks carbon-oxygen bonds such as those found in the common β-O-4 interunit linkage. The resulting deconstruction products contain a mixture of aromatic monomers and oligomers, with the oligomers primarily containing the remaining 5-5, β-1, β-5, and β-β carbon-carbon interunit linkages. For example, analysis of RCF lignin oil derived from pine wood identified components that collectively accounted for 57% of the oil by weight, of which 34% were monomers, 16% dimers, and 7% trimers (7). While chemical conversion of complex oligomer mixtures is difficult (8), microbes routinely assimilate carbon from mixed feedstocks and can be engineered to funnel that carbon to desired products (9–12).

The simplest approach for lignin valorization by biological funneling involves combining key pathways for assimilation of diverse aromatic compounds into a production host such as *Pseudomonas putida* KT2440, *Parasphingobium lignivorans* SYK-6 (formerly *Sphingobium lignivorans* SYK-6, [13, 14]), or *Novosphingobium aromaticivorans* F199 (hereafter “KT2440,” “SYK-6,” and “F199”) (15–17). These strains vary in their ability to catabolize aromatic oligomers (Fig. 1): *P. putida* has not been shown to natively catabolize any model lignin-associated aromatic dimers; SYK-6 can natively catabolize GGE (18), DDVA (19), DGPD (20), and DCA (21); while F199 natively catabolizes GGE (22, 23), DGPD (24), and DCA (25). However, none of these strains can natively catabolize all of the monomeric and oligomeric compounds commonly found in deconstructed lignin, motivating the discovery and heterologous expression of additional catabolic pathways.

The majority of pathways for catabolism of lignin-associated aromatic dimers have been characterized in SYK-6 and F199 (Table 1), though additional pathways have recently been discovered in other bacteria (26–28). A comparison of these pathways in sphingomonads highlights parallel biochemical solutions for cleavage of diverse lignin-associated aromatic dimers. In contrast, the one published example of a dimer catabolic pathway in a pseudomonad demonstrates an entirely different biochemical logic (28, 29). This review will first summarize known catabolic pathways for lignin-associated aromatic dimers and then highlight biochemical parallels between the pathways. Potential explanations for these parallels will be discussed, as well as the potential to test hypotheses through pathway expression and optimization in heterologous hosts.

**TABLE 1:** Homologs of lignin catabolism genes in *P. lignivorans* SYK-6, *N. rhizosphaerae* LY, and *N. aromaticivorans* F199

Gene name	Annotation	SYK-6 locus tag	LY homolog locus tag	F199 homolog locus tag
*ligD*	NAD(P)^+^-dependent dehydrogenase (short-chain alcohol dehydrogenase family)	SLG_08640	None found	SARO_RS01025 Saro_0205
*ligE*	β-Etherase	SLG_08660	R9J51_10665	SARO_RS12100 Saro_2405
*ligF*	Glutathione *S*-transferase family protein	SLG_08650	R9J51_11700	SARO_RS10520 Saro_2091
*ligG*	Glutathione *S*-transferase	SLG_08670	None found	None found
*ligL*	NAD(P)^+^-dependent dehydrogenase (short-subunit alcohol dehydrogenase family)	SLG_33660	None found	SARO_RS09390 Saro_1875
*ligN*	NAD(P)^+^-dependent dehydrogenase (short-subunit alcohol dehydrogenase family)	SLG_35900	None found	SARO_RS03965 Saro_0794
*ligO*	NADP-dependent 3-hydroxy acid dehydrogenase YdfG	SLG_35880	None found	SARO_RS03960 Saro_0793
*ligP*	β-Etherase	SLG_32600	R9J51_10665	None found
*ligQ*	Glutathione-dependent disulfide-bond oxidoreductase	SLG_04120	R9J51_19385	SARO_RS13080 Saro_2595
*hpvZ*	HPV oxidase	SLG_12830	None found	SARO_RS19130 Saro_3651
*ldpA*	*erythro*-DGPD Cγ-formaldehyde lyase	SLG_12650	R9J51_00435	SARO_RS14230 Saro_2805
*ldpB*	DGPD-keto Cα reductase	SLG_12640	R9J51_00440	SARO_RS03515 Saro_0703
*ldpC*	DGPD-keto Cα reductase	SLG_12690	R9J51_00335	SARO_RS07815 Saro_1560
*ligW2*	5-Carboxyvanillate decarboxylase	SLG_12810	R9J51_00160	SARO_RS08370 Saro_1670
*ligW*	5-Carboxyvanillate decarboxylase	SLG_07850	None found	SARO_RS03990 Saro_0799
*ligXa*	5,5′-Dehydrodivanillate *O*-demethylase oxygenase subunit	SLG_07770	None found	None found
*ligXc*	5,5′-Dehydrodivanillate *O*-demethylase ferredoxin subunit	SLG_08500	R9J51_18420	None found
*ligXd*	5,5′-Dehydrodivanillate *O*-demethylase ferredoxin reductase subunit	SLG_21200	R9J51_03850	None found
*ligY*	OH-DDVA meta-cleavage compound hydrolase	SLG_07750	None found	None found
*ligZ*	OH-DDVA oxygenase	SLG_07720	None found	None found
*bzaA*	Aromatic aldehyde dehydrogenase	SLG_27910	R9J51_21665	SARO_RS12605 Saro_2503
*phcC*	DCA-C oxidase	SLG_09480	None found	None found
*phcD*	DCA-C oxidase	SLG_09500	None found	None found
*phcF*	DCA-CC decarboxylase	SLG_09360	None found	None found
*phcG*	DCA-CC decarboxylase	SLG_09370	R9J51_15745	None found
*pcfL*	γ-formaldehyde lyase	None found	None found	SARO_RS03975 Saro_0796
*pinZ*	Pinoresinol reductase	SLG_07320	R9J51_00450	SARO_RS14245 Saro_2808
*pinY*	Lariciresinol oxidase	SLG_27980	R9J51_15355	None found
*pinX*	Lariciresinoate decarboxylase	SLG_07290	R9J51_00385	SARO_RS14590 Saro_2877
*pinW*	Imperanene oxidase	None found	R9J51_00375	SARO_RS14585 Saro_2876
*pinV*	Cytochrome *c*_6_	None found	R9J51_00370	SARO_RS14580 Saro_2875
*ferA*	Feruloyl-CoA ligase	SLG_25020	R9J51_02980	SARO_RS03365 Saro_0674
*ferB*	*p*-Hydroxycinnamoyl CoA hydratase/lyase	SLG_25030	R9J51_10560	SARO_RS08365 Saro_1669
*ferD*	5-formylferulate dehydrogenase	SLG_12800	R9J51_00170	SARO_RS03980 Saro_0797
*desV*	Aromatic aldehyde dehydrogenase	SLG_28320	R9J51_00415	SARO_RS19370 Saro_3700
*ligV*	Vanillin dehydrogenase	SLG_07060	R9J51_10555	SARO_RS08360 Saro_1668
*desA*	Syringate *O*-demethylase	SLG_25000	R9J51_10670	SARO_RS12095 Saro_2404
*desC*	Alpha/beta hydrolase	SLG_12720	R9J51_00325	SARO_RS14525
*desD*	Glutathione-*S*-transferase	None found	R9J51_00330	SARO_RS14530 Saro_2865
*ligH*	Formate-tetrahydrofolate ligase	SLG_12760	None found	None found
*ligM*	Aminomethyl transferase family protein	SLG_12740	R9J51_00310	SARO_RS14510 Saro_2861
*metF*	Methylenetetrahydrofolate reductase	SLG_12750	R9J51_00305	SARO_RS14505 Saro_2860
*desB*	Gallate dioxygenase	SLG_03330	None found	None found
*desZ*	3-*O*-Methylgallate 3,4-dioxygenase	SLG_19030	None found	None found
*gdmA*	Guaiacol demethylase	None found	None found	SARO_RS07455 Saro_1487
*ligA*	Protocatechuate 4,5-dioxygenase, α subunit	SLG_12510	R9J51_00480	SARO_RS14270 Saro_2813
*ligB*	Protocatechuate 4,5-dioxygenase, β subunit	SLG_12500	R9J51_00475	SARO_RS14265 Saro_2812
*ligC*	4-Carboxy-2-hydroxymuconate-6-semialdehyde dehydrogenase	SLG_12490	R9J51_00470	SARO_RS14260 Saro_2811
*ligI*	2-Pyrone-4,6-dicarboxylate hydrolase	SLG_12570	R9J51_00510	SARO_RS14275 Saro_2814
*ligJ*	2-Keto-4-carboxy-3-hexenedioate hydratase	SLG_12520	R9J51_00485	SARO_RS14300 Saro_2819
*ligK*	4-Carboxy-4-hydroxy-2-oxoadipate aldolase	SLG_12550	R9J51_00500	SARO_RS14290 Saro_2817
*ligR*	LysR family transcriptional regulator	SLG_12540	R9J51_00495	SARO_RS14285 Saro_2816
*ligU*	4-Oxalomesaconate tautomerase	SLG_12560	R9J51_00505	SARO_RS14295 Saro_2818
*lsdA*	Carotenoid oxygenase family protein	SLG_12580	None found	SARO_RS14250 Saro_2809
*lsdB*	Carotenoid oxygenase family protein	SLG_09440	None found	None found
*lsdC*	Carotenoid oxygenase family protein	SLG_11300	None found	None found
*lsdD*	Carotenoid oxygenase family protein	SLG_12860	R9J51_00140	SARO_RS04005 Saro_0802
*lsdE*	Carotenoid cleavage dioxygenase	SLG_27300	None found	None found
*lsdF*	Carotenoid oxygenase family protein	SLG_27970	R9J51_00455 (*pinU*)	None found
*lsdG*	Carotenoid oxygenase family protein	SLG_36640	None found	None found
*lsdH*	Carotenoid oxygenase family protein	SLG_37540	None found	None found

## PATHWAYS FOR CATABOLISM OF AROMATIC DIMERS WITH β-1 LINKAGES

**Fig 2:**
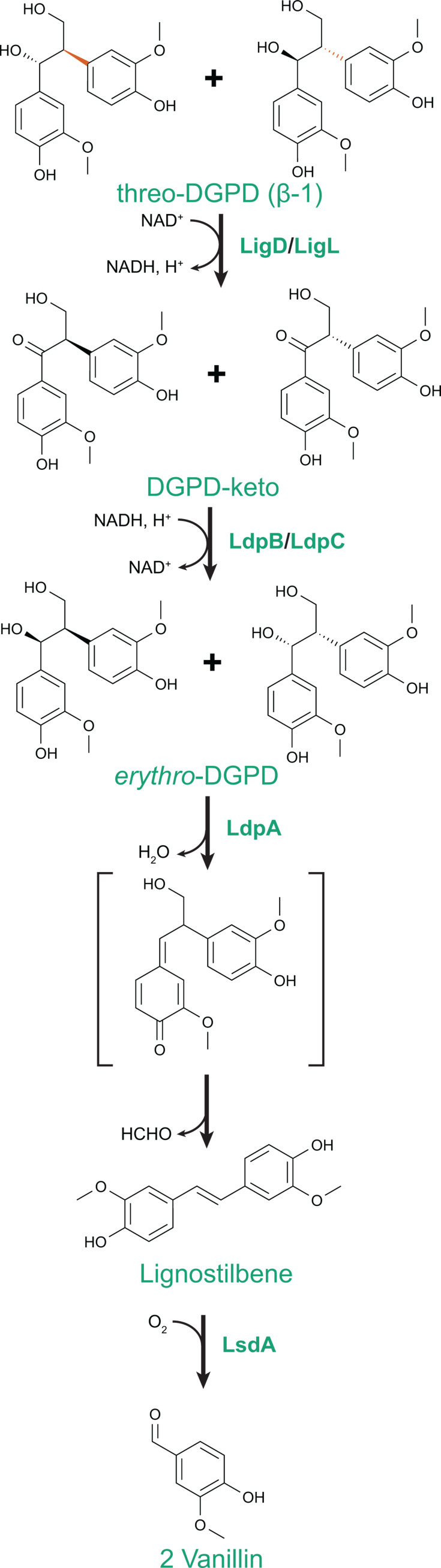
Catabolic pathway for DGPD, a model aromatic dimer with a β-1 linkage. Homologs of LigD, LigL, LdpB, and LdpC have been characterized from SYK-6. Homologs of LdpA and LsdA have been characterized from SYK-6 and F199.

Diguaiacylpropanediol (DGPD) is a model aromatic dimer with a β-1 linkage that can be formed by acid-catalyzed ring-opening of a spirodienone linkage in lignin (Fig. 2) (30). A pathway for *erythro*-DGPD catabolism was biochemically characterized in *Sphingobium paucimobilis* TMY1009 (31, 32), but the gene for the first enzyme was not cloned. An enzyme that performs a similar transformation was later identified and characterized in F199 (24). A homologous pathway was then demonstrated in SYK-6 (20) and further expanded to encompass catabolism of *threo*-DGPD (33).

The two *threo*-DGPD isomers are first stereoselectively oxidized to the corresponding Cα carbonyl analog by LigD and LigL, both members of the short-chain dehydrogenase/reductase (SDR) family (33). The Cα carbonyl analogs are then stereoselectively reduced to the corresponding *erythro*-DGPD isomers by the SDR-family reductases LdpB and LdpC. These four enzymes are in the same enzyme family but demonstrate strict selectivity for substrate stereochemistry and reaction directionality.

The *erythro*-DGPD isomers can then be converted to lignostilbene by an enzyme from the NTF2 superfamily, LdpA (previously termed LsdE) (20). This enzyme is strictly selective for *erythro*-DGPD but demonstrates equivalent kinetics with both *erythro* diastereomers. While an enzyme was identified in *S. paucimobilis* TMY1009 that catalyzes a similar reaction (31), differences between the reported molecular weight and oligomerization with those of LdpA (20) suggest that the TMY1009 deformylase may not be closely related to LdpA.

Finally, lignostilbene is oxidatively cleaved by a lignostilbene dioxygenase to yield two equivalents of vanillin (34–37). SYK-6 contains eight lignostilbene dioxygenase homologs with varying substrate specificity, several of which are active with lignostilbene (38, 39). F199 has two lignostilbene dioxygenases (40, 41) that appear to be functionally redundant during growth with DGPD (24).

## PATHWAYS FOR CATABOLISM OF AROMATIC DIMERS WITH β-5 LINKAGES

**Fig 3:**
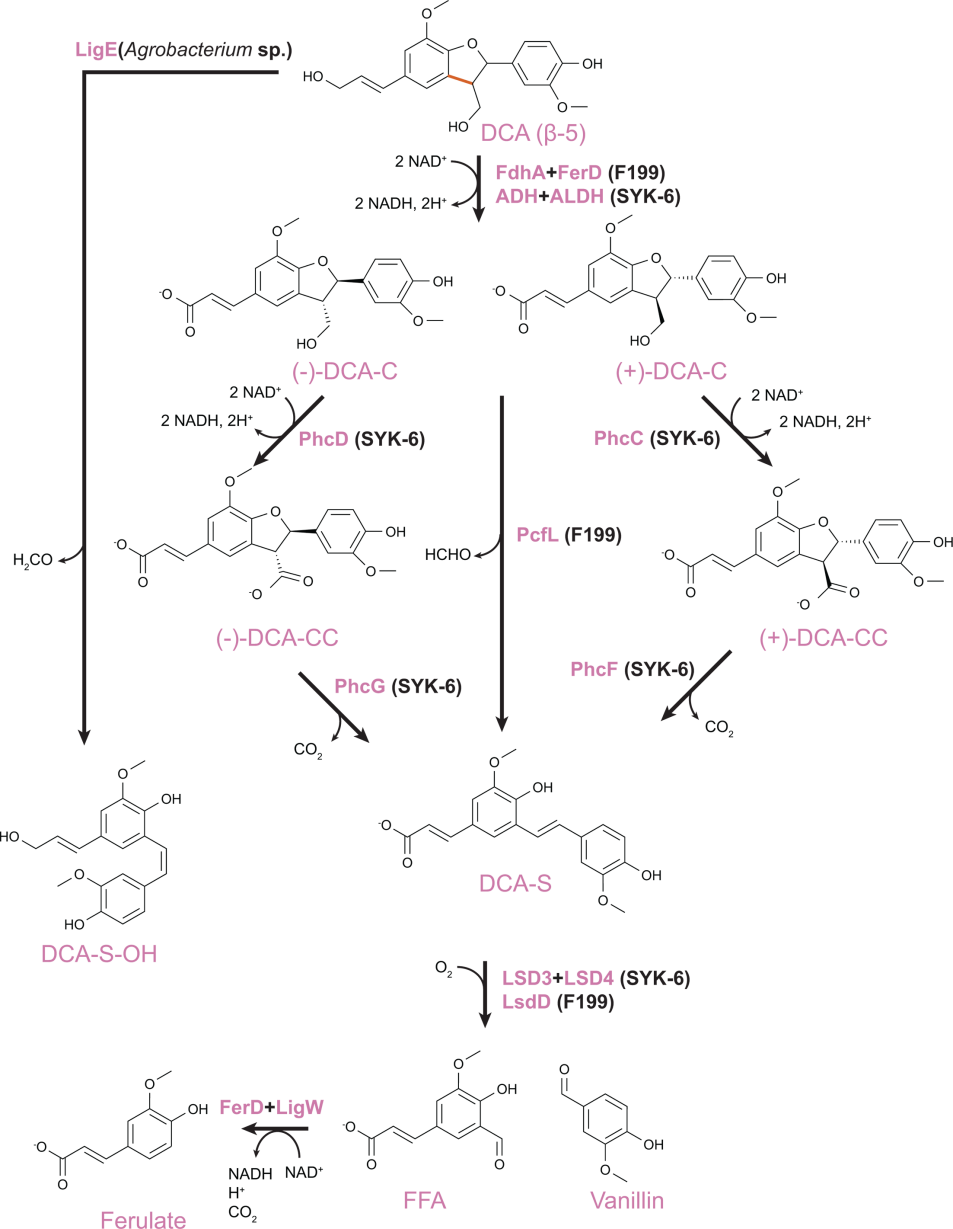
Catabolic pathway for DCA, a model aromatic dimer with a β-5 linkage. The enzyme source is indicated.

Dehydrodiconiferyl alcohol (DCA) is a model aromatic dimer with a β-5 linkage (Fig. 3). The catabolic pathway for this compound was first described in SYK-6 (21). In SYK-6, isomers of DCA are first oxidized to DCA-C by a range of promiscuous oxidases and then stereoselectively oxidized to DCA-CC by PhcC or PhcD (42). DCA-CC is then stereoselectively decarboxylated by PhcG or PhcF to yield a stilbene, DCA-S (43). This stilbene can be oxidatively cleaved by a lignostilbene dioxygenase to generate vanillin and 5-formylferulate (38, 39).

Recently, equivalent pathways for catabolism of 5-formylferulate have been described in both SYK-6 and F199 (25, 44). In both organisms, 5-formylferulate is first oxidized to 5-carboxylferulate by an aldehyde dehydrogenase FerD, followed by decarboxylation to ferulate catalyzed by the LigW and LigW2 decarboxylases (45, 46).

However, further investigations into DCA catabolism in other bacteria have uncovered catabolic pathways that vary in key reactions. In F199, oxidation of DCA to DCA-C is similar to the SYK-6 pathway, but both DCA-C isomers are then directly converted to DCA-S with loss of formaldehyde through the action of PcfL, a homolog of LdpA from the β-1 pathway (25). Four stereoselective enzymes, PhcCDFG, are effectively replaced with a single nonselective enzyme. However, the trade-offs between these two approaches have not yet been investigated.

In a third example, a glutathione transferase from an *Agrobacterium* species, termed LigE, was shown to convert DCA to a stilbene analog of DCA-S, also with loss of formaldehyde (26). The biological context of this LigE-catalyzed deformylation has not yet been established, for example, testing for further conversion of the stilbene intermediate DCA-S-OH either to DCA-S or directly to vanillin and 5-formyl coniferyl alcohol.

## PATHWAYS FOR CATABOLISM OF AROMATIC DIMERS WITH β-β LINKAGES

Recently, a pathway for catabolism of the lignan (+)-pinoresinol, which contains a β-β interunit linkage, was described in a new bacterial isolate, termed *Novospingobium rhizosphaerae* LY (hereafter “LY”) (27). The first step in pinoresinol catabolism, reductive cleavage of a furan ring by PinZ to yield lariciresinol, had been proposed based on studies in SYK-6 (47). However, SYK-6 was unable to fully assimilate the resulting products, so a complete reaction pathway could not be identified.

**Fig 4:**
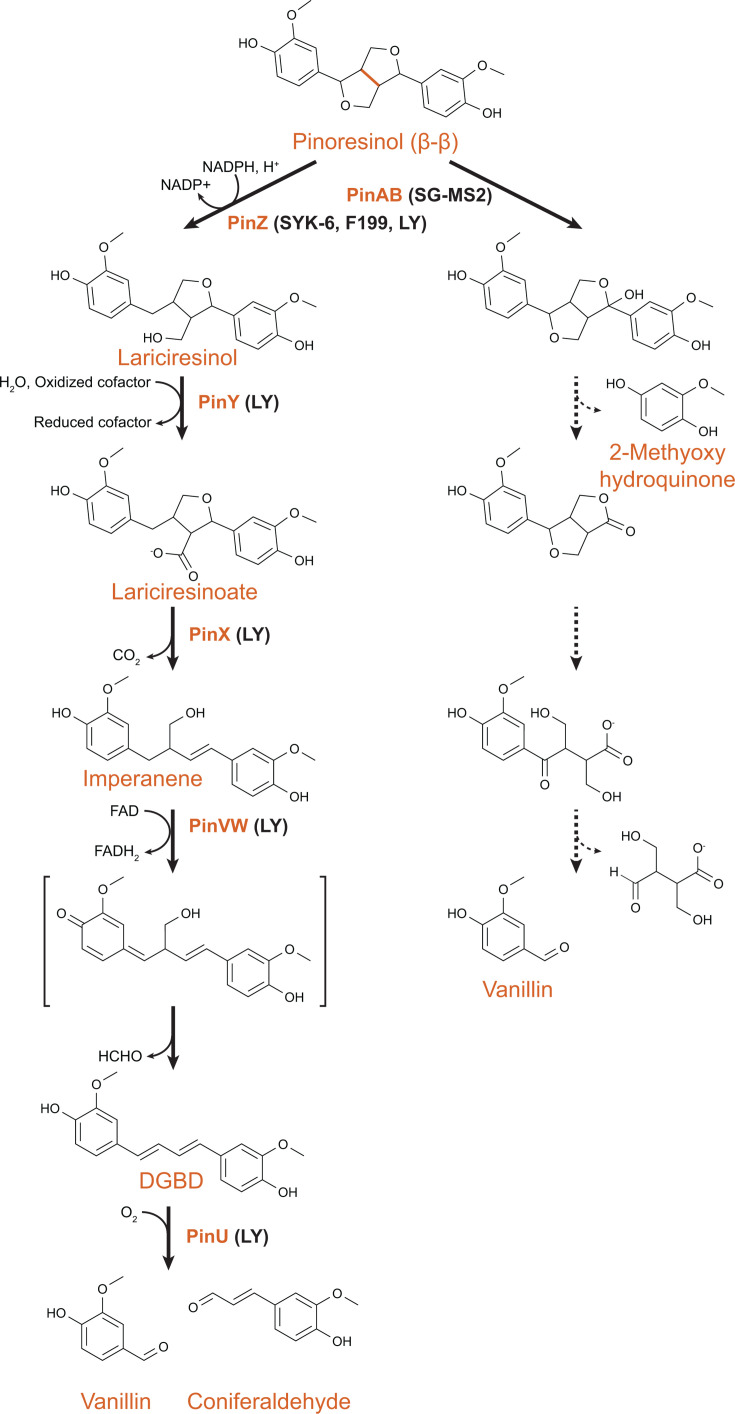
Catabolic pathway for pinoresinol, a model aromatic dimer with a β-β linkage. Homologs of PinZ have been characterized in SYK-6, F199, and LY. The first reaction in pinoresinol catabolism in SG-MS2 has been characterized, with further characterization needed on the later reactions.

As predicted, the pinoresinol catabolic pathway from LY begins with a PinZ-catalyzed reductive ring opening and then proceeds through a combination of biochemical transformations previously described in both the β-1 and β-5 pathways (Fig. 4). Reductive cleavage of a furan ring by PinZ yields lariciresinol, which is then oxidized by PinY to lariciresinoate. Next, the PinX decarboxylase, which is a distant homolog of PhcG and PhcF from β-5 catabolism in SYK-6, catalyzes a decarboxylation to open the remaining furan ring and introduce a double bond in the aromatic linker. The flavocytochrome PinVW then oxidizes imperanene to a proposed quinone methide intermediate, followed by loss of formaldehyde to yield diguaiacylbutadiene (DGBD). DGBD resembles an extended lignostilbene and similarly can be oxidatively cleaved by PinU, a homolog of lignostilbene dioxygenase. PinU-catalyzed cleavage of DGBD yields vanillin and coniferyl aldehyde, which can be metabolized through previously characterized pathways.

A pathway for catabolism of (+)-pinoresinol was partially characterized in the Gammaproteobacterium *Pseudomonas* sp. SG-MS2 (28, 29). While details of this pathway remain to be validated, the pathway is clearly distinct from that described in strain LY. Pinoresinol degradation is initiated by the PinA hydrolase, a member of the VAO/PCMH flavoprotein protein family, in combination with a cotranslationally-expressed cognate PinB cytochrome (29). Cleavage of the hydroxylated pinoresinol by a putative *ipso*-hydroxylase would yield two aromatic monomers that could ultimately be assimilated through extensions of known pathways.

## OTHER PATHWAYS FOR CATABOLISM OF LIGNIN-RELATED AROMATIC DIMERS

Extensive prior work has established core pathways for catabolism of guaiacylglycerol-β-guaiacyl ether (GGE), a model aromatic dimer with a β-O-4 linkage, and dehydrodivanillate (DDVA), a model dimer with a 5-5 linkage (48, 49). Additional pathway details continue to be elucidated, including for GGE catabolism the details of ether bond cleavage (22, 50, 51), regulation (52), and assimilation of the resulting aromatic monomers (23, 53–55)*,* and for DDVA the key role of transporters in catabolism (56, 57). Transporters have not been implicated in catabolism of the other lignin-associated aromatic dimers described above, likely because they are uncharged at physiological pH (58).

In nature, lignin is oxidatively deconstructed to low-molecular-weight products using a variety of laccase and peroxidase enzymes, primarily by fungi though with minor contributions from bacteria (59, 60). The extent of deconstruction and the fate of the resulting products vary based on strain, biomass source, and environmental conditions (3, 4). When oligomeric intermediates are generated, oxidative enzymes such as laccases and peroxidases can potentially cleave the oligomers into monomers (61). However, oxidative cleavage competes with spontaneous repolymerization of radical intermediates (3, 62). Recent work has also identified pathways for assimilation of lignin-associated aromatic monomers by fungi (63). These fungi can convert 4-hydroxybenzoate to hydroxyquinol, which is then assimilated by way of β-ketoadipate (64). This pathway is biochemically analogous to that found in *Rhodococcus jostii* RHA1 (65), though the enzymes involved are not closely related. Specific pathways for fungal degradation of model aromatic compounds, similar to those described above for bacteria, have not yet been reported. The abundance of such pathways in bacteria suggests that oligomers are commonly produced in the environment but primarily degraded by bacteria. Bacterial assimilation may benefit the fungi by reducing lignin repolymerization and relieving product inhibition (66, 67).

## PROMISCUITY AND PARALLEL BIOCHEMICAL EVOLUTION

In combination, seven partial or complete pathways for catabolism of aromatic dimers with β-1, β-5, and β-β linkages have been described (Fig. 2 to 4). In comparing these pathways, biochemical parallels can be observed. Six pathways, primarily from sphingomonads, generate stilbene intermediates that can be oxidatively cleaved to yield monomeric aromatic aldehydes. The seventh pathway, for pinoresinol catabolism by a pseudomonad, follows a very different biochemical logic from the other six. In this proposed pathway, the two aromatic moieties are separated at the beginning of the pathway, and these monomers are then further transformed into common catabolic intermediates such as vanillin.

**Fig 5:**
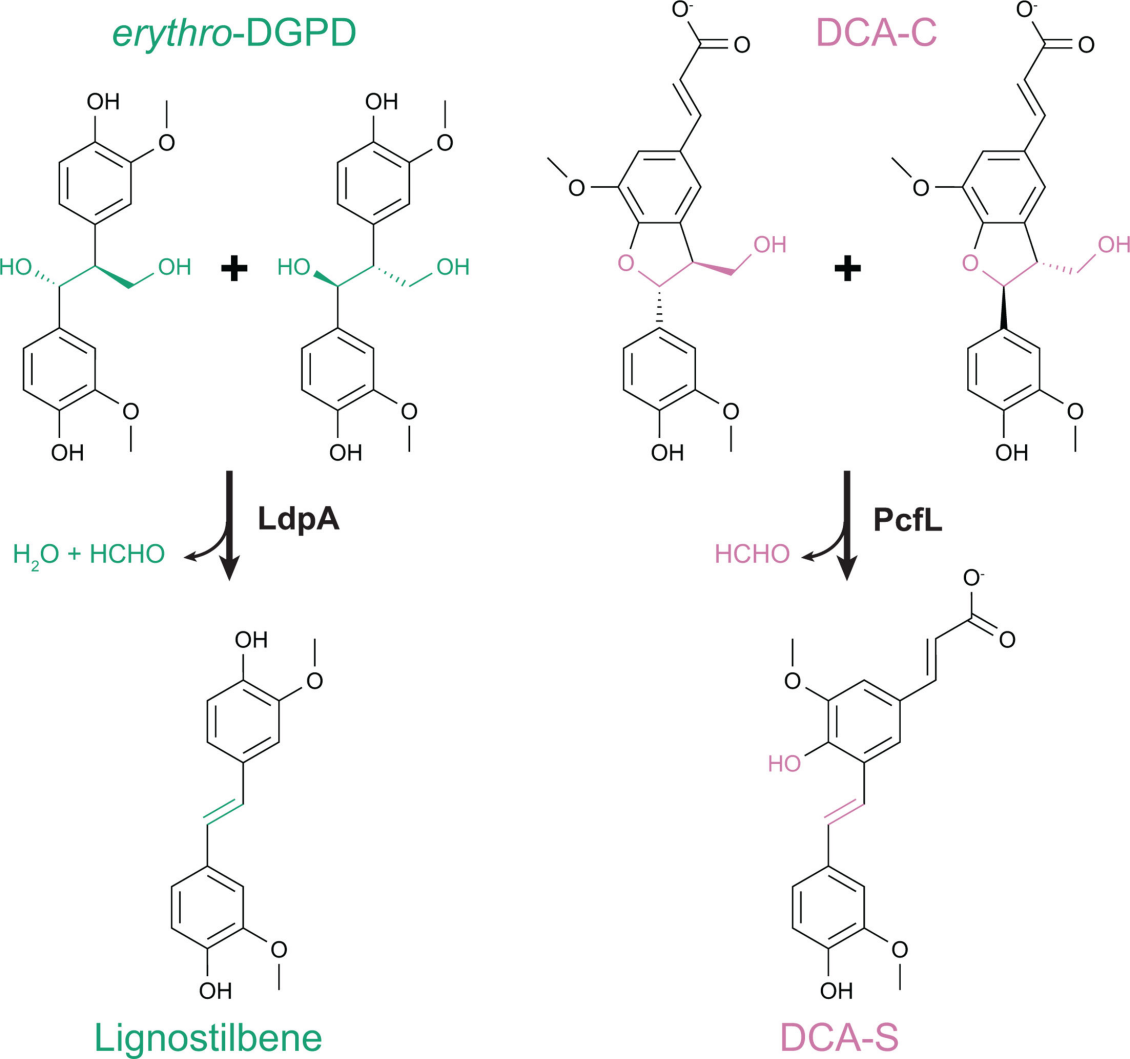
Similar deformylation reactions are observed in catabolism of aromatic dimers with β-1 and β-5 bonds, both in *N. aromaticivorans*. Equivalent moieties for deformylation are highlighted in green (DGPD) and pink (DCA-C).

**Fig 6:**
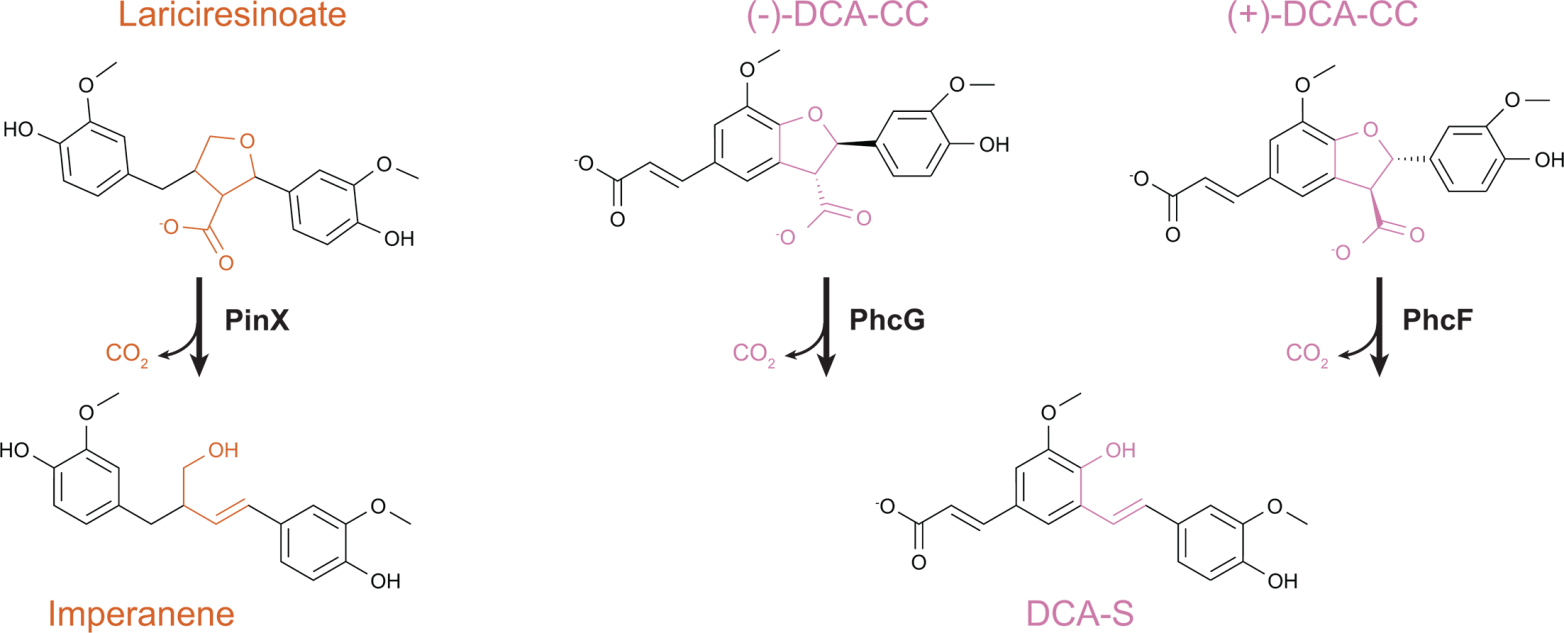
Similar decarboxylation reactions are observed in catabolism of aromatic dimers with β-β and β-5 bonds. Equivalent moieties for decarboxylation are highlighted in orange (lariciresinol) and pink (DCA-CC).

The six stilbene-generating pathways employ multiple parallel routes to stilbene formation. In some cases, homologous enzymes catalyze similar reactions using different substrates in different pathways. Two enzymes from the NTF2 family, LdpA and PcfL, catalyze similar stilbene-forming deformylations but using different substrates, the β-1-linked *erythro*-DGPD and the β-5-linked DCA-C, respectively (Fig. 5) (24, 25). Similarly, homologous decarboxylases PhcF/PhcG and PinX introduce double bonds with loss of CO_2_ in the β-5-linked DCA-CC and β-β-linked lariciresinoate, respectively (Fig. 6) (43).

**Fig 7:**
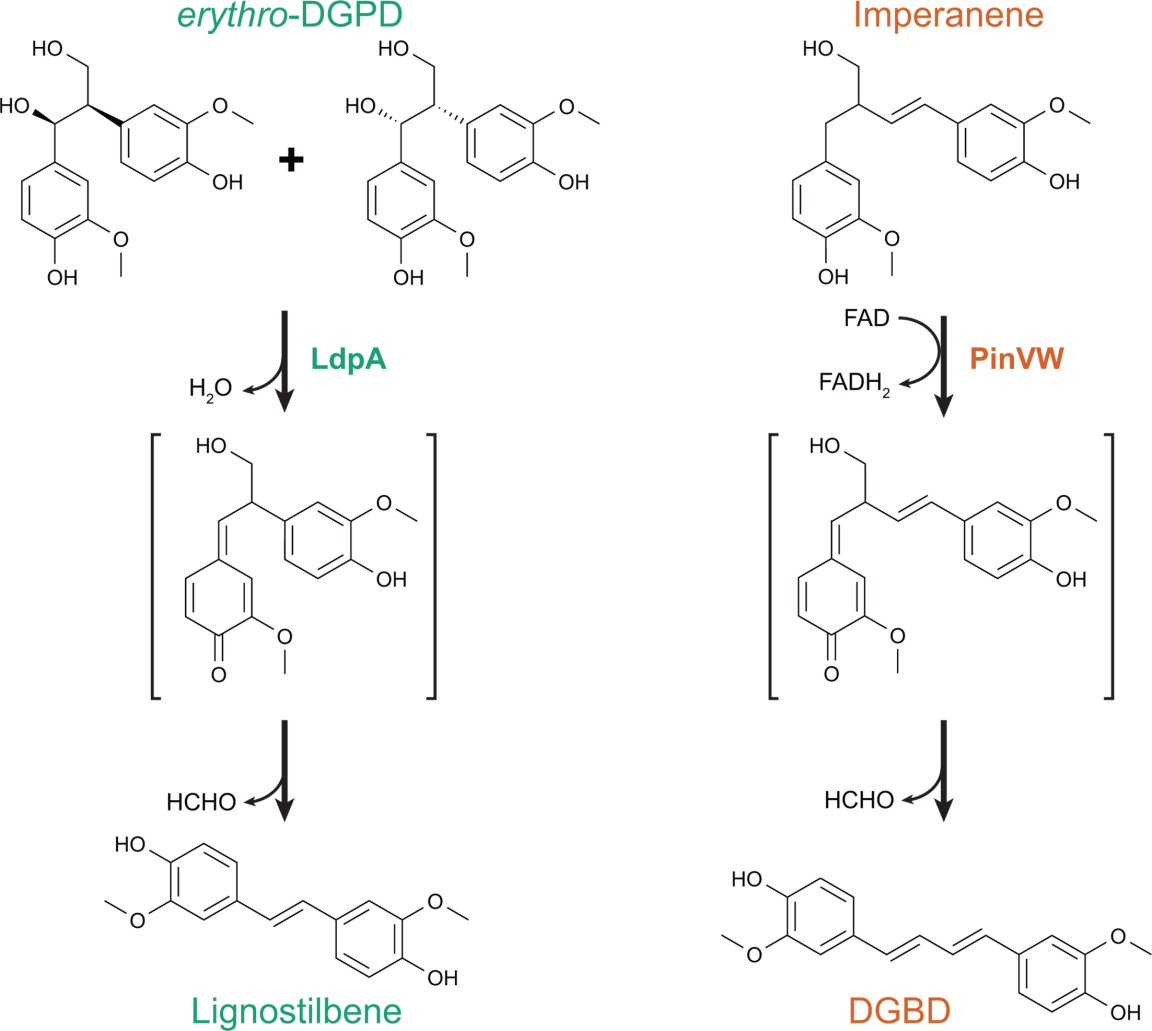
Parallel deformylations in aromatic dimer catabolism are catalyzed by different enzyme classes. LdpA is a member of the NTF2 superfamily and catalyzes dehydration to form a quinone methide intermediate. PinVW is a member of the cresol dehydrogenase family and catalyzes an oxidation to form a similar quinone methide.

In other examples, however, unrelated enzymes perform similar transformations on related substrates. In DCA catabolism, stilbene formation can be catalyzed by stereoselective PhcF/PhcG decarboxylases or the non-stereospecific NTF2 family deformylase PcfL and glutathione-*S*-transferase LigE (Fig. 3). While the details of the reaction chemistry vary, in all three cases, an intermediate quinone methide is likely formed. It remains to be seen whether similar alternatives exist in other pathways. For example, the transformation of lariciresinol to imperanene catalyzed by PinXY is similar to that catalyzed by PhcCDFG and could potentially be replaced by a homolog of PcfL. Similarly, a quinone methide intermediate has been proposed for the reaction of LdpA with *erythro*-DGPD and PinVW with imperanene (20, 27). In both cases, the intermediate is proposed to resolve through the loss of formaldehyde that introduces a double bond in the linker between aromatic rings. However, the first half-reaction differs between the two enzymes, with LdpA catalyzing a dehydration to form the quinone methide intermediate and PinVW an oxidation (Fig. 7).

Finally, there is evidence of substrate promiscuity simplifying the assimilatory networks. For example, LigW and LigW2 decarboxylate 5-carboxyvanillate from DDVA catabolism to yield vanillate (45, 46) and decarboxylate 5-carboxyferulate from DCA catabolism to yield ferulate (25, 44). Similarly, LigL and related Cα-dehydrogenases oxidize both GGE and *threo*-DGPD (33, 68). Homologous enzymes from the lignostilbene dioxygenase family have been shown to cleave diverse stilbene intermediates (38, 39). It is likely that other members of these enzyme families have evolved to catabolize additional lignin-associated aromatic compounds. While the substrate specificity of these enzymes is unknown, the number of unique homologs gives a sense of the scale involved. For example, SYK-6 has eight lignostilbene dioxygenase homologs and 62 homologs from the NTF2 superfamily. Some of these enzymes are likely to be involved in pathways unrelated to assimilation of lignin-associated aromatic compounds, but additional lignin-associated pathways undoubtedly await characterization.

This enzyme promiscuity may explain many of the biochemical parallels noted above. New enzyme activities are thought to most frequently evolve from promiscuous side activities through a process of innovation, amplification, and divergence (69–71). If enzyme homologs in one dimer catabolic pathway can easily evolve to catalyze similar reactions with a different substrate, then pathways will tend to follow a similar progression of enzymatic transformations due to evolutionary rather than biochemical factors. Conversely, convergent evolution of similar transformations catalyzed by independently evolved enzymes suggests that the underlying biochemistry is the primary driving factor. The examples summarized above suggest that both mechanisms have been important in the evolution of dimer catabolic pathways.

Additionally, epistatic interactions with the host or other endogenous biochemical pathways may bias pathway evolution. A strain with a high capacity to express a particular class of enzymes or tolerate specific toxic intermediates may preferentially evolve pathways using those enzymes or generating those intermediates. Assessing this last factor will require additional experiments.

## HETEROLOGOUS EXPRESSION AND OPTIMIZATION OF AROMATIC CATABOLIC PATHWAYS

Since no known bacterium catabolizes the full range of potential lignin-associated aromatic compounds, efficient biological funneling will likely require assembly of multiple heterologous pathways in a single production host. In addition to its value for improved lignin valorization, heterologous pathway expression provides an opportunity to directly test hypotheses about catabolic pathway assembly. If pathway assembly has largely been driven by evolutionary contingency and biochemical feasibility, then pathways would work equally well in different genetic contexts. Alternatively, if epistatic interactions are major factors, then certain host/pathway combinations will be more productive.

However, few conclusions can yet be drawn, since relatively few catabolic pathways for lignin-associated aromatic compounds have been expressed in any heterologous host and generally only one pathway has been tested at a time. The complete pathway for catabolism of *erythro*-DGPD was successfully transferred from F199 to KT2440. KT2440 natively catabolizes vanillin, so expression of LdpA and LsdA was sufficient to stoichiometrically convert DGPD into vanillin. In this example, the resulting vanillin was ultimately converted into a bioproduct, *cis,cis*-muconate, rather than used as directly as a growth substrate (24). Though not a focus of this review, a portion of the pathway for DDVA catabolism has been transferred from SYK-6 to KT2440 (72). Heterologous expression of LigXacd, LigY, LigZ, DdvK, and DdvT enabled conversion of DDVA to 5-carboxyvanillate with a yield of approximately 30%.

Direct comparisons of pathways in native and heterologous hosts are difficult to interpret, since the native hosts have previously evolved to optimize pathway function. Similarly, effective growth using a heterologous pathway has often required experimental evolution to optimize activity or expression. For example, an endogenous pathway for GGE catabolism in F199 has also been optimized using experimental evolution (23). Serial propagation with GGE as the sole carbon source identified several mutations that improved growth yield, primarily through deregulation of native pathways that presumably were not induced under laboratory conditions. Simultaneous expression of multiple pathways will likely require additional engineering to improve pathway modularity and portability, minimizing interactions between co-expressed heterologous pathways and between pathways and the host organism (73).

As a result, most engineering efforts have focused on transferring pathways for assimilation of aromatic monomers. These efforts provide useful lessons that could inform studies using dimer catabolic pathways. For example, two pathways for guaiacol catabolism have been independently expressed in KT2440 or related strains: the cytochrome P450 monooxygenase GcoAB from *Amycolatopsis* sp. ATCC 39116 (74, 75) or *Rhodococcus rhodochrous* J3 (76) and the Rieske monooxygenase GdmAB from *Cupriadivus necator* N-1 (53). Since KT2440 natively catabolizes catechol, expression of only the guaiacol demethylase was sufficient to enable growth. GcoAB was first expressed in *Acinetobacter baylyi* ADP1. Serial propagation of the resulting strain selected for a fusion between the catechol dioxygenase CatA and the guaiacol demethylase GcoA. Chromosomal expression of this fusion protein, but not the unfused enzymes, enabled growth of KT2440 with guaiacol as a sole carbon source (74). Similarly, expression in *P. putida* EM42 of a GcoA homolog from *R. rhodochrous* on a medium-copy plasmid enabled growth with guaiacol as a sole growth substrate, albeit with a long lag phase (76). Conversely, chromosomal expression of GdmAB in KT2440 enabled rapid growth with guaiacol (53), perhaps due to more efficient production of Rieske-type monooxygenases in this host. In the case of dimers with only one known catabolic pathway, additional bioprospecting could identify pathways that are more readily transferred, for example, those with evidence of prior horizontal gene transfer (77).

Similarly, two unrelated pathways for *O*-demethylation of vanillate have been described, exemplified by the Rieske monooxygenase VanAB from KT2440 (78) and the tetrahydrofolate-dependent methyltransferase LigM from SYK-6 (79). When VanAB in KT2440 was replaced with LigM, growth with vanillate required co-expression of MetF and LigH to enhance tetrahydrofolate cycling as well as a mutation to the host that likely also affected single-carbon metabolism (80). Additional experimental evolution of the LigM-expressing KT2440 further improved growth, but an optimized VanAB-expressing strain outperformed the optimized LigM strain (80). Understanding interactions between heterologous and endogenous pathways could enable better pathway selection and more straightforward design of combinatorial metabolic networks.

Heterologous pathways for assimilation of protocatechuate have been expressed in KT2440 (81) and *Escherichia coli* (82). In KT2440, the native protocatechuate 3,4-cleavage pathway was replaced with the seven-gene 4,5-cleavage pathway from SYK-6. This heterologous pathway was active and could be used to improve the yield of target bioproducts, though direct growth comparisons are difficult to assess. Similarly, the 3,4-cleavage pathway from KT2440 has been transferred into *E. coli*. Chromosomal expression of the nine-gene *pca* pathway was sufficient to enable growth with protocatechuate. However, experimental evolution with protocatechuate identified additional mutations that increased both expression of the protocatechuate dioxygenase PcaH and the growth rate with protocatechuate (82). Using this improved strain, expression of the *hcaABC* pathway from *A. baylyi* ADP1 or the *couLMNO* pathway from *R. jostii,* in addition to further experimental evolution, was sufficient to enable growth with coumarate as a sole growth substrate (83). The additional mutations identified through experimental evolution included mutations to both heterologous and endogenous genes, demonstrating that heterologous pathway expression can impose new stresses on the expression host, for example, by inhibiting an essential endogenous biosynthetic enzyme (83). These deleterious interactions are likely to multiply as additional pathways are expressed in a production host and will need to be alleviated to enable high productivity.

## CONCLUSION AND FUTURE DIRECTIONS

Lignin catabolism is thought to have evolved approximately 300 million years ago (84), giving ample time for pathway evolution. Perhaps unsurprisingly, multiple biochemical pathways have evolved independently to assimilate lignin-associated aromatic compounds, exemplified by the different enzymes used in DCA catabolism by SYK-6 and F199 or the unrelated pathways for pinoresinol catabolism in LY and SG-MS2. Similarly, independently evolved enzymes and pathways have been identified for catabolism of aromatic monomers such as protocatechuate (65, 85–88), catechol (85, 89), guaiacol (53, 74), and vanillate (78, 79). Recent discoveries suggest that additional pathways remain to be identified, even for well-studied substrates like DCA and pinoresinol. Characterizing these pathways and understanding the tradeoffs will further illuminate the underlying biochemical logic of aromatic oligomer catabolism and provide additional resources for lignin valorization by bacterial metabolic engineering. For example, bioinformatic evidence suggests that some bacteria may have parallel pathways for DCA catabolism, using homologs of both PhcF/PhcG and PcfL (25). If these parallel pathways are demonstrated, under what circumstances does redundancy provide an advantage over a single pathway?

Much of the pathway discovery described above has considered the role of dimer stereochemistry but focused on dimers with two guaiacyl (G) moieties. However, many lignin degradation products will likely contain hydroxycinnamoyl (H) and syringyl (S) moieties. Select reactions have been tested with a panel of related substrates, for example, the glutathione-*S*-transferases LigE/LigF/LigP with guaiacylglycerol-β-guaiacyl ether (GGE), guaiacylglycerol-β-syringyl ether (GSE), syringylglycerol-β-guaiacyl ether (SGE), and syringylglycerol-β-syringyl ether (SSE) (90); the LdpA deformylase with DGPD and 1,2-dihydroxyphenyl-1,3-propanediol (DHPD) (24); and the PinZ reductase with pinoresinol (GG), syringaresinol (SS), medioresinol (SG), and ligballinol (HH). In general, analysis of dimer degradation pathways is limited by the availability of suitable model compounds, and dimers with varied monomers and defined stereochemistry are even more challenging to synthesize or source commercially. Increasing availability of diverse model dimers would enable a more comprehensive analysis of the substrate promiscuity of aromatic catabolic enzymes and thereby identify missing reactions that would motivate further discovery.

Finally, metabolic engineering efforts for complex assimilatory pathways are still in the early stages, primarily exemplified by transfer of short pathways for assimilation of monomeric aromatic compounds. Engineering strains with optimized combinations of pathways, particularly for multi-gene oligomer assimilation pathways, will require new techniques for pathway regulation to minimize deleterious interactions and pathway optimization to alleviate those that still remain. It is not yet clear the degree to which interactions between assimilatory pathways tend to be beneficial or deleterious. If they are generally beneficial, then strains such as SYK-6 or F199 that natively contain multiple pathways for aromatic oligomer assimilation will be good hosts for further engineering. If instead the interactions are deleterious, then assembly of a community of specialist strains would be more advantageous. Additional research is needed to identify the best path forward.
